# Targeting myocardial ischaemic injury in the absence of reperfusion

**DOI:** 10.1007/s00395-020-00825-9

**Published:** 2020-10-14

**Authors:** M. V. Basalay, D. M. Yellon, S. M. Davidson

**Affiliations:** The Hatter Cardiovascular Institute, 67 Chenies Mews, London, WC1E 6HX UK

**Keywords:** Ischaemia, Heart, Cardioprotection, Animals, Infarction, Remodelling, Regeneration

## Abstract

Sudden myocardial ischaemia causes an acute coronary syndrome. In the case of ST-elevation myocardial infarction (STEMI), this is usually caused by the acute rupture of atherosclerotic plaque and obstruction of a coronary artery. Timely restoration of blood flow can reduce infarct size, but ischaemic regions of myocardium remain in up to two-thirds of patients due to microvascular obstruction (MVO). Experimentally, cardioprotective strategies can limit infarct size, but these are primarily intended to target reperfusion injury. Here, we address the question of whether it is possible to specifically prevent ischaemic injury, for example in models of chronic coronary artery occlusion. Two main types of intervention are identified: those that preserve ATP levels by reducing myocardial oxygen consumption, (e.g. hypothermia; cardiac unloading; a reduction in heart rate or contractility; or ischaemic preconditioning), and those that increase myocardial oxygen/blood supply (e.g. collateral vessel dilation). An important consideration in these studies is the method used to assess infarct size, which is not straightforward in the absence of reperfusion. After several hours, most of the ischaemic area is likely to become infarcted, unless it is supplied by pre-formed collateral vessels. Therefore, therapies that stimulate the formation of new collaterals can potentially limit injury during subsequent exposure to ischaemia. After a prolonged period of ischaemia, the heart undergoes a remodelling process. Interventions, such as those targeting inflammation, may prevent adverse remodelling. Finally, harnessing of the endogenous process of myocardial regeneration has the potential to restore cardiomyocytes lost during infarction.

## Introduction

**Fig. 1:**
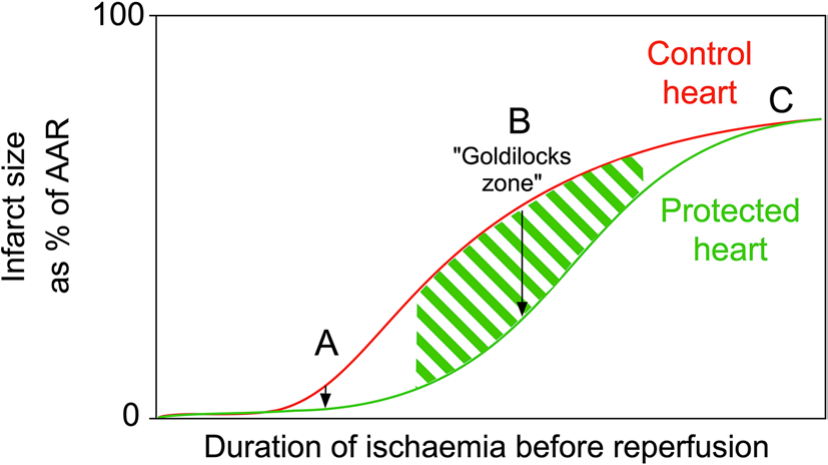
The potential for cardioprotective strategies to limit myocardial infarction depends on the duration of ischaemia prior to reperfusion. If the duration of ischaemia before reperfusion is very short (**a**), then even if the relative reduction in infarct size is significant, the absolute reduction in infarct size will be very small. If the duration of ischaemia is excessively long (**c**), cardioprotective strategies will be ineffective. After intermediate durations of ischaemia followed by reperfusion (the “goldilocks” zone) (**b**), protection is maximal. Note that in this example, infarct size (as a percentage of ischaemic area at risk or AAR) is measured at a point after reperfusion, and accounts for cell death occurring during both ischaemia and reperfusion

An acute coronary syndrome results in a sudden reduction of blood flow to the myocardium. In the case of ST-elevation myocardial infarction (STEMI), this is usually caused by the acute rupture of atherosclerotic plaque and thrombotic obstruction of a coronary artery. Non-STEMI (NSTEMI) may be precipitated by coronary artery spasm or other factors that restrict blood flow. Reperfusion therapy, with the aim of rapidly returning blood flow to the ischaemic myocardium, has been a key advance in the treatment of STEMI. If blood flow in the culprit coronary artery is not promptly restored, the obstruction inevitably leads to myocardial death. The size of myocardial infarction (MI) is known to be a major determinant of prognosis in these patients (reviewed in [56]). Timely restoration of blood flow in the culprit coronary artery(s) is able to reduce infarct size (IS) in patients presenting with STEMI, and has become the first-line therapeutic strategy. However, reperfusion causes further injury itself, called reperfusion injury [47, 55, 56]. Reperfusion-induced death of cardiomyocytes, or lethal myocardial reperfusion injury, may account for up to 50% of the final IS [47]. Furthermore, even when recanalisation is successful, ischaemic regions of myocardium remain in up to 2/3 of patients due to microvascular obstruction (MVO), which is associated with a larger IS, a lower left ventricle (LV) ejection fraction, adverse LV remodelling, and worse clinical outcomes [47, 53, 56]. Experimentally, cardioprotective strategies can further limit IS, so long as they are applied prior to or close to the onset of reperfusion [6, 47, 55, 56]. Ischaemic preconditioning (IPC) is considered the gold standard of cardioprotection. Classically, in the first description of IPC in 1986, Murry et al. showed that IPC limited IS in dogs when they were subject to 40 min myocardial ischaemia followed by reperfusion, but not when the duration of ischaemia was extended to 3 h [90]. This pivotal publication clearly shows that following excessive duration of myocardial ischaemia, there is little or no opportunity for IS reduction (point C in Fig. 1). On the other hand, if the ischaemic period is very short, the resulting infarcted area can be so small that any IS reduction achieved with a putative cardioprotective intervention on top of reperfusion therapy may not translate into a clinical benefit (point A in Fig. 1) [10, 56]. Only in the “goldilocks” zone (point B in Fig. 1), where ~ 30–70% of the area at risk (AAR) is infarcted, is there the possibility for cardioprotection to be both significant and clinically meaningful [38].

The majority of studies since Murry et al.’s seminal paper have, therefore, focussed on trying to limit IS in experimental models in which a moderate duration of 30–60 min ischaemia is followed by reperfusion [45]. The focus of these studies has been to specifically target reperfusion injury, since if a drug must be administered prior to ischaemia, it is already too late for the patient who presents with STEMI. However, perhaps this focus on reperfusion injury is only part of the story, and there could be benefit in targeting ischaemic injury itself [24]. For one thing, delaying the injurious process during ischaemia might allow prolongation of the window during which reperfusion therapy can be successfully applied. Furthermore, ischaemic myocardium may be a viable target in other clinical settings, such as rescue PCI, NSTEMI or MINOCA (Myocardial Ischaemia with Non-Obstructive Arteries). Finally, regions of ischaemic myocardium can persist following reperfusion when MVO occurs. For example, in a pig model, MVO was apparent early after reperfusion, peaking at 24 h when it reached 6.5% of left ventricle, and progressively decreasing thereafter [31]. Interestingly, a number of publications claim to detect a reduction of IS by cardioprotective strategies in animal models of permanent coronary artery ligation, in which there is myocardial ischaemia *without* any reperfusion whatsoever [26, 27, 29, 67, 69, 84, 89, 105]. Can this be true? Is it possible to specifically target injury caused by ischaemia alone? Here, we critically examine the evidence for a potential therapeutic benefit of cardioprotective strategies designed to target non-reperfused myocardium.

## The mechanism of ischaemic injury

First, it is important to understand the process that occurs in cardiomyocytes during ischaemia. In the complete absence of blood supply, residual oxygen in the ischaemic zone of the myocardium is depleted within seconds. Glycolysis is able to produce sufficient ATP for basal cell activity for a limited time, but the kinetics of this reaction pathway are gradually overwhelmed by the accumulation of its reaction products (lactate and protons), slowing down ATP production. Without oxygen, mitochondria in the ischaemic zone are no longer able to produce ATP. However, the mitochondrial membrane potential is maintained by the F_0_F_1_-ATPase acting in reverse, pumping protons out of the mitochondria while hydrolysing ATP to ADP. This is a major contributing factor to the further precipitous reduction in cellular ATP. Around 20–60 min ischaemia, ATP is completely depleted, cardiomyocytes undergo ischaemic contracture (shortening and stiffening), and sarcolemmal ion pumps cease to function. The inability to remove calcium ions from the cell then results in catastrophic calcium overload and oncotic cell death, which is defined by irreversible sarcolemmal disruption and necrosis [23]. Therefore, after a certain duration of *complete* ischaemia, there is no intervention that can conceivably salvage ischaemic myocardium—even reperfusion—as the cardiomyocytes are already lethally damaged. A necrotic cardiomyocyte cannot be brought back to life.

The precise time at which cardiomyocytes start to die varies across the heart, because cells in different regions are exposed to ischaemia for different durations of time. Due to the relatively high pressure on vessels in the subendocardium, ischaemia occurs initially in this region. Cardiomyocytes exhaust their ATP stores and undergo ischaemic contracture, thereby increasing pressure in adjacent myocardium and causing the region of ischaemia to progress towards the epicardium. This results in the typical “wavefront” of infarct progression as initially described by Reimer and colleagues [101]. On the other hand, cardiomyocytes that are exposed to low residual oxygen levels may be able to sustain sufficient ATP levels to survive for an extended period, even if the ATP is insufficient to enable their contraction.

**Fig. 2:**
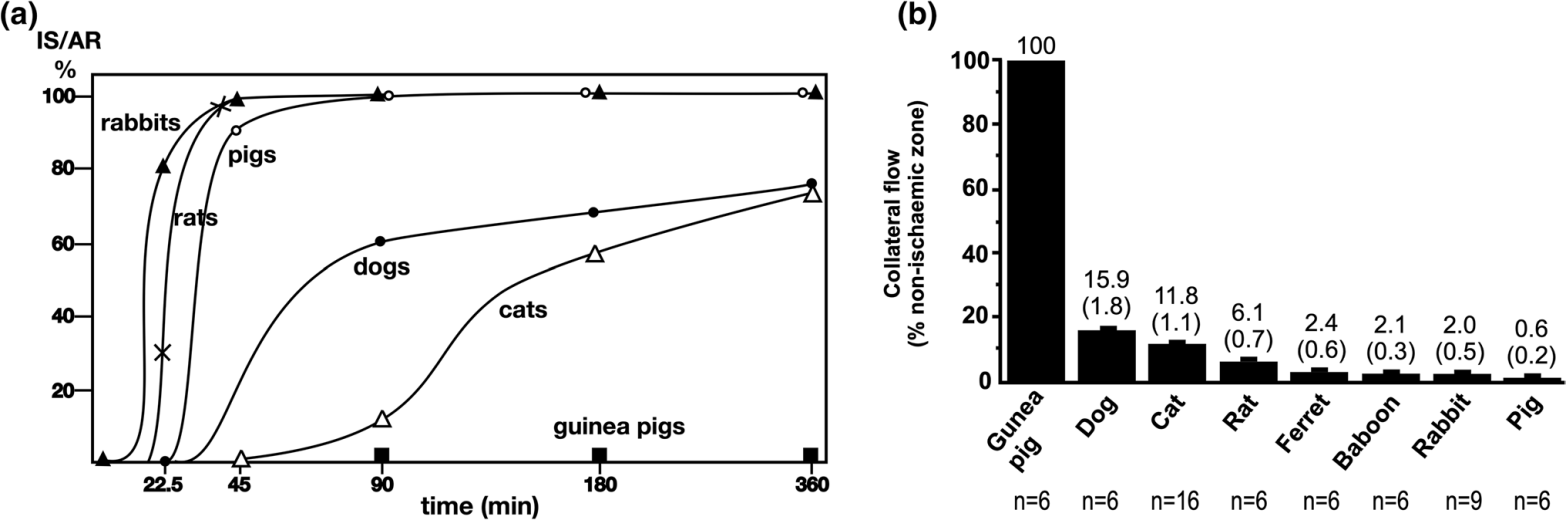
a Infarct size expressed as a percentage of area at risk, vs time after coronary occlusion in rabbits, rats, pigs, dogs, cats and guinea pigs. Infarcts develop rapidly and completely in rats, rabbits and pigs. Redrawn from Schaper W et al. Prog Cardiovasc Dis. 1988; 31:57–77, with permission from Elsevier. **b** Collateral flow in different species, as a percentage of flow in the ischaemic vs non-ischaemic myocardium (mean ± SEM). *n* = number of hearts examined. Redrawn from Maxwell MP et al., Cardiovasc Res. 1987; 21(10):737–746, with permission from Oxford University Press

Interestingly, the rate of IS progression after coronary artery occlusion (CAO) varies markedly between species (Fig. 2a). Nevertheless, at some time point, the infarct region inevitably reaches an upper size limit that is typical for each animal species. In mice, rats, rabbits and pigs, ~ 90% of the area at risk (AAR) is infarcted after only 45–90 min of CAO [109, 111]. After 90 min ischaemia in dogs, however, the IS is only ~ 60% of the AAR, and even after 6 h, reaches a maximum of only ~ 70% [109, 111]. The explanation for the myocardial resistance in dogs is their relatively high number of coronary collateral vessels, which can maintain a residual flow of blood to ischaemic myocardium (Fig. 2b) [48, 86]. Consequently, there is a close negative correlation between subepicardial collateral blood flow and IS [70]. Remarkably, guinea pigs have such abundant collateral circulation in their hearts that they do not show any infarction even after 24 h of CAO [48, 109, 111]. It should also be noted that the extent of coronary collateralization varies not only between species, but also between different breeds of the same species [48]. For example, coronary collateral flow in studies using dog models varies from 2% to around 40% [48]. In addition, collateral blood flow can differ between different coronary vascular territories within the same heart [8]. The importance of residual flow on the progression of MI is clearly demonstrated by experiments using low-flow ischaemia instead of complete coronary occlusion. For example, infarction progresses rapidly in pigs, which have minimal coronary collateralization, but no infarction is seen even after 12 h of moderate myocardial ischaemia [114]. In human hearts, the extent of collateralization varies markedly between individuals. In addition, many other factors affect the rate of IS progression in clinical trials, as opposed to experimental studies. However, myocardial IS in STEMI patients typically does not reach its maximum value until 12 h or longer [93, 113].

## Animal models of myocardial ischaemia

Despite years of study, cardioprotective strategies that are effective in animal models have failed to translate to clinical trials [51]. Various limitations of animal models that may explain this disconnect have been discussed, but one major difference is in the duration of ischaemia. In animal experiments, a period of 30–60 min ischaemia followed by reperfusion is widely used [11]. Does this accurately model the human STEMI patient for whom the aim is to administer reperfusion therapy within 12 h of symptom onset? A recent study of first‐time STEMI patients with an occluded culprit coronary artery (TIMI flow 0), single‐vessel disease, and no coronary collaterals (Rentrop 0)—ie.: in whom *complete* myocardial ischaemia was likely—found that myocardial salvage index was low after a symptom-to-balloon time of more than 2 h [41]. By 2 h, there was already a high degree of transmurality in 84% patients examined by MRI, with the infarct area extending across the full width of the myocardium. Importantly, the transmural extent of infarction is the most important predictor of LV remodelling and clinical outcomes, and is independent from overall size of infarction in predicting cardiac prognosis in the longer term [2]. Therefore, in the absence of significant collateralization or residual flow, there is very little myocardium remaining to salvage after only 2 h, and cardioprotective strategies may be futile after this time. Analysis of multiple clinical trials in patients with different Rentrop scores suggests a slightly extended time frame for optimal reperfusion therapy, by showing that percutaneous coronary interventions in patients presenting 2–3 h after symptom onset can provide substantial improvement in myocardial salvage, while further delay leads to a rapid reduction of the volume of viable myocardium [38]. On the other hand, if collateral flow is sufficient to slow infarct development, a novel cardioprotective strategy might still be effective in patients who are reperfused after as long as 12 h ischaemia [93, 113]. In this case, however, the appropriate animal model to investigate cardioprotection might be one with collaterals or residual low-flow ischaemia followed by reperfusion. As most commonly used rodent and pig models of acute coronary artery occlusion are characterized by a nearly complete absence of myocardial collateral circulation, it is worth considering whether they are the most appropriate model for this group of patients [86].

## Measuring infarct size in ischaemic models

Even if an intervention is able to limit acute ischaemic injury in a model lacking collateral vessels, the effect could be challenging to measure. The gold standard method of determining IS in experimental settings is to incubate myocardial slices in a dye, such as triphenyltetrazolium chloride (TTC) [11, 79]. When reduced by dehydrogenases present in the tissue, TTC turns into a distinct brick-red colour, clearly indicating the area of live myocardium [11]. A crucial factor is that a sufficiently long period of reperfusion (at least 1–2 h) at a reasonable flow rate is required to wash out enzymes completely from dead cells. If not, dead tissue can artefactually appear red (i.e. false positive). In other words, a period of reperfusion is necessary to “reveal” the infarct using tetrazolium staining. A similar problem is presented by other techniques that estimate IS indirectly via the release of troponins or lactate dehydrogenase (LDH) in the perfusate/blood, since flow is necessary to wash them out of dead cells. A further important consideration is that after a long duration of ischaemia, the infarcted myocardium may not be well reperfused due to the “no reflow” phenomenon (MVO), which will result in an artefactual red-stained region of dead tissue following tetrazolium staining. This effect may explain the observation in Langendorff isolated perfused heart experiments, that a long period of global ischaemia can result in a paradoxically smaller infarct region. However, this hypothesis has not yet been carefully investigated. Interestingly, an early study in dogs found that TTC staining was effective at detecting infarcted myocardium after coronary occlusion without reperfusion, as all infarcted regions coincided with areas showing histologic features of early ischaemic infarction [91]. Notably, however, dogs are often characterised by an abundant system of collaterals in the myocardium, which may expedite the wash-out of enzymes from the infarcted area.

Cardiac magnetic resonance imaging (MRI) can be useful as it allows evaluation not only of the function of myocardium, but also visualization of its detailed structure including the infarcted area. The technique to detect the infarcted area is known as Late Gadolinium Enhancement (LGE) and is similar in the clinical setting and in animal studies [19]. LGE requires administration of chelated gadolinium, to increase contrast between the viable and hyper-enhanced infarcted myocardium. It has been shown to be an early marker of irreversible injury and eventual fibrosis, as the enhanced area matches the infarct defined by TTC [122, 124]. However, the use of LGE technique in the model of CAO is hampered by the same problem as TTC staining. Whereas with TTC staining, it is unclear how the enzymes can be washed out from the dead cells if the culprit artery is occluded, with LGE the occluded artery can hinder the contrast agent from entering the infarcted area. In dogs, which have extensive collateralization, comparison of ex vivo MRI to TTC-stained sections demonstrated that the area of LGE reflected the area of infarction throughout the period of infarct healing, from 4 h to 8 weeks, regardless of reperfusion status [32, 98]. On the other hand, in pigs, which have few collaterals, no delayed hyperenhancement was found 4 h after permanent coronary artery occlusion [124]. However, after 8 h, LGE appeared and its spatial extent gradually increased until it became transmural at 8 days [124]. Furthermore, 4 days after the induction of non-reperfused MI in pigs, IS determined by LGE was in excellent agreement with IS measured using TTC staining [30]. The only possible mechanism for contrast agent uptake by completely non-reperfused myocardium is slow interstitial diffusion from the surrounding tissues that are still well-supplied [124]. The border zone of the MI, which is usually composed of a mixture of viable and nonviable myocytes, can already be distinguished by the contrast agent in the first minutes after intravenous administration, presumably by contrast coming from intact collaterals [121]. Interestingly, the reperfused and the non-reperfused MIs in pigs were shown to accumulate contrast agent at a similar rate. The latter observation may confirm the hypothesis that most of the contrast uptake is through slow interstitial diffusion in both MI models [30]. Although MRI allows accurate evaluation of infarct volume and infarct shrinkage with the course of time, the accuracy of this in chronic models of coronary occlusion in animals without coronary collaterals requires further investigation.

Another simple and widely available method to detect ischaemic injury in non-reperfused myocardium in an experimental setting is electrocardiography (ECG). Although the sensitivity of ECG for detecting acute MI in humans is quite low [85, 120], ST elevation in most animal models can already be seen minutes after the left anterior descending coronary artery occlusion. This makes ECG a valuable tool to observe the effects of cardioprotective agents on the progression of myocardial ischaemia. For example, in studies conducted in pigs, IPC and remote IPC did not affect initial ST-segment elevation, but attenuated ST-segment elevation after 55 min ischemia. Importantly, this effect was not due to altered blood flow into the ischaemic region [3, 71]. The mechanism for this is not known but could potentially involve the parasympathetic nervous system, which is required to convey the signal of remote IPC, and is known to innervate the heart [6]. The shortcomings of ECG are that it does not allow accurate measurement of IS, especially in small animal species, and it probably reflects a combination of both the ischaemic AAR and IS.

## Strategies to reduce the rate of ischaemic injury

Assuming that it is possible to identify a way to accurately measure cell survival in non-reperfused myocardium, what interventions could conceivably delay infarct development in the scenario of chronic ischaemia? Based on the classical concept, that the fate of myocardial tissue in the face of an obstructed coronary artery depends on the balance between myocardial oxygen supply and demand [12], two types of intervention could be envisaged: those that reduce myocardial oxygen consumption and those that increase oxygen/blood supply. Either of these should slow ATP depletion and delay the “wavefront” of necrosis. Although such a delay cannot prevent eventual infarction if reperfusion is not achieved, it may be advantageous in extending the window during which reperfusion can be successfully performed.

### Strategies to reduce myocardial oxygen consumption

A reduction in myocardial oxygen consumption occurs as part of an innate physiological mechanism called “myocardial hibernation”, in which myocardial contractile activity is suppressed. This allows the heart to match myocardial energy and substrate metabolism to a severe reduction in regional coronary flow [58]. The restoration of myocardial energy balance can protect the heart from ischaemia, though only for a limited time. Short-term hibernation explains the survival of some myocardium that can be rescued in acute STEMI patients even after 24 h from symptom onset (reviewed in [54]).

Various techniques have been proposed as able to reduce myocardial oxygen demand during ischaemia. For example, hypothermia [5, 21]; cardiac unloading [88]; the reduction of heart rate, contractility or systolic wall tension [12]; and IPC [44, 90] appear able to slow infarct development in animal models. Dunker et al. showed a linear relationship between core body temperature and IS in pigs subjected to 45-min myocardial ischaemia and reperfusion, such that, at 35 °C, no infarction occurred, and with each 1 °C increase in temperature an additional 20% of the area at risk became infarcted [28]. Importantly, a patient-level, pooled analysis of six randomized trials of endovascular cooling during primary percutaneous coronary intervention (PCI) in STEMI, including 629 patients, showed a significant reduction in IS in patients with anterior STEMI who were cooled to < 35 °C [20].

**Fig. 3:**
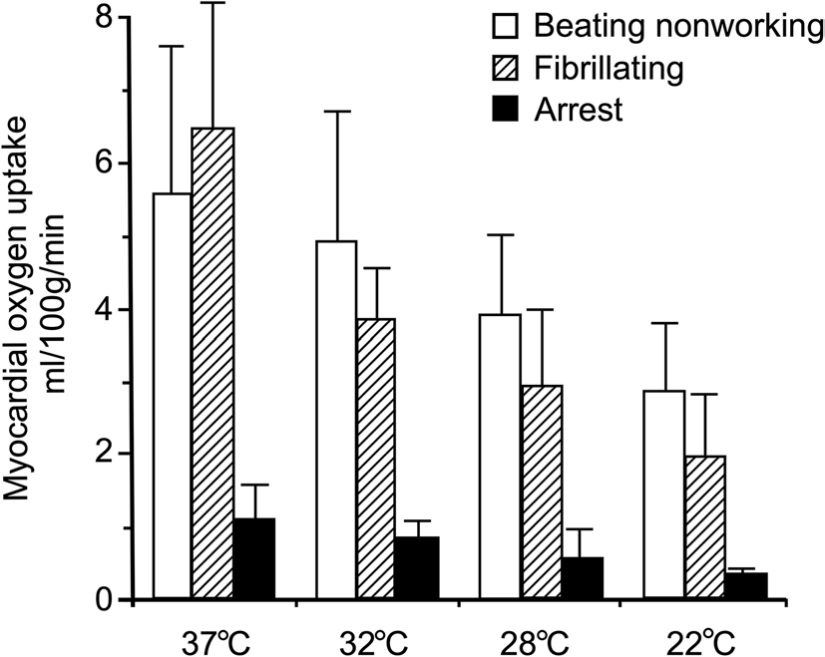
Myocardial oxygen uptake during cardiopulmonary bypass at different temperatures ranging from 37 to 22 °C. Redrawn from Buckberg GD. J Thorac Cardiovasc Surg. 1991; 102:895–903, with permission from Elsevier

In fact, temperature has a relatively minor effect on myocardial energy requirements, and the principal determinant is the amount of cardiac electromechanical work being performed [15]. A substantial temperature reduction to 22 °C is required to even halve oxygen demand, whereas normothermic arrest (i.e., at 37 °C) reduces oxygen demand by ~ 90% (Fig. 3) [15]. These data come from healthy myocardium, and it is less clear that ischaemic tissue, which does not contract, would benefit by having contraction work further inhibited. On the other hand, in one trial in 187 patients with STEMI treated with primary PCI, heart rate at presentation was negatively associated with myocardial salvage index [4], which suggests that electromechanical work can be a contributing factor to IS in patients.

**Fig. 4:**
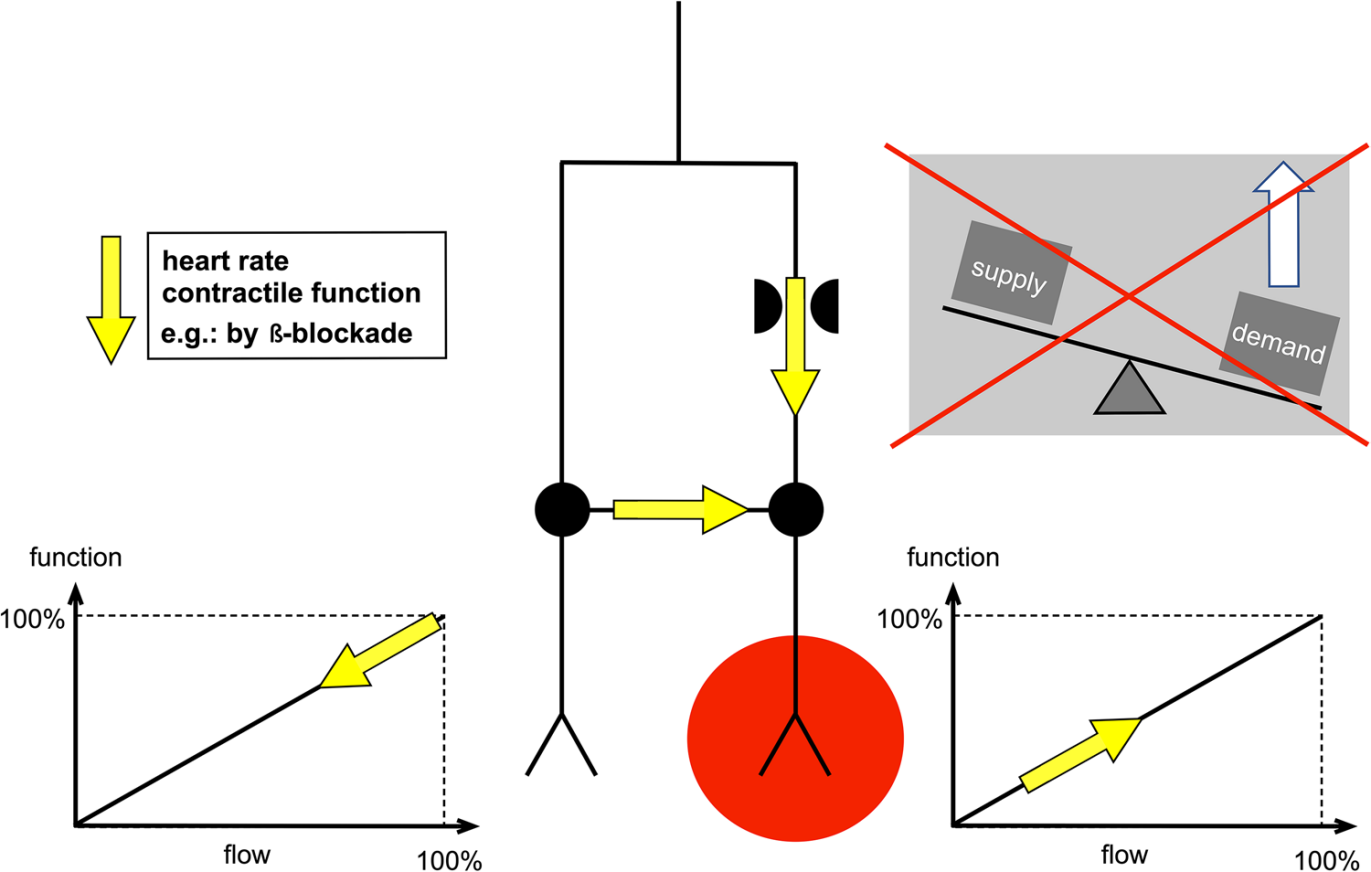
Redistribution of coronary blood flow between ischaemic and non-ischaemic myocardium by beta-blockade. Reprinted from Heusch G. Am J Physiol Heart Circ Physiol. 2019; 316:H1439-H1446, with permission from G Heusch

The potential benefit of a reduction in heart rate and cardiac work on IS has been extensively investigated. Experiments in sheep [88] and dogs [1, 104], as well as recent human data [118], demonstrated that LV mechanical unloading with a non-surgical heart pump during the acute phase of MI strikingly reduces IS. Maroko et al. were the first to show that when haemodynamic determinants of myocardial oxygen consumption were decreased using the beta-blocker propranolol, ST-segment elevation and creatine kinase leakage during 24 h coronary artery occlusion were reduced [84]. Conversely, catecholamines, which increase the haemodynamic determinants of myocardial oxygen consumption, caused an increase in IS in dogs subject to coronary artery occlusion [84]. This attenuation of regional myocardial ischaemia can be explained by a redistribution of blood flow. In the non-ischaemic region, beta-blockade reduces contractile function, and coronary flow reduces to match the metabolic demand. At the same time, the ischaemic myocardium receives more blood flow through the stenosis and through collaterals (Fig. 4) (reviewed in [54]). Unfortunately, however, most subsequent experiments failed to confirm the benefit of beta-blockers in reducing IS, either following prolonged ischaemia or after IR [49, 60]. In 2013, some excitement was generated by the METOCARD-CNIC trial in which intravenous metoprolol reduced IS following STEMI [61]. Interestingly, the earlier metoprolol was administered during infarction (i.e. ischaemia), the smaller was the IS [36]. Unfortunately, the subsequent EARLY-BAMI trial of early intravenous metoprolol before PPCI saw no reduction in IS, casting doubt on previous conclusions [103].

In experimental settings, cardioprotection has been achieved with ivabradine [59, 72], an inhibitor of the pacemaker I_f_ channel, known mostly as a heart rate reducing medication. However, its infarct-limiting effect is proposed to be realized by several other mechanisms, unrelated to heart rate [16], and remains be confirmed in clinical trials.

Another intuitively appealing and well-studied approach is to use calcium channel blockers to limit infarct development via decreasing oxygen consumption. Indeed, a verapamil infusion decreased IS in dogs after 24 h CAO, and this benefit was sustained until 48 h [69]. However, clinical studies found that calcium channel blockers neither reduced myocardial IS nor improved survival (reviewed in [73]). A possible explanation is that any potential cardioprotective benefit of cardio-depressants is likely to be counteracted by the corresponding reduction in systemic blood pressure, resulting in undesirable blood flow redistribution away from the heart.

Despite the possible benefit of these drugs in delaying the progression of infarction, one has to consider that no compound can enter an ischaemic no-flow area of myocardium, particularly if the culprit coronary artery in totally occluded. For this reason, any drug intended to directly affect the ischaemic area would have to be administered before the onset of the ischaemia. An alternative approach is to employ a treatment that can work remotely to protect the ischaemic myocardium. For example, electrical stimulation of the vagal nerves, which innervate the heart, preserved myocardial creatine kinase depletion in dogs after 24 h permanent coronary ligation [105]. This most likely functions through a reduction in heart rate and LV function, as well as an increase in coronary blood flow through a nitric oxide (NO)-dependent mechanism. However, vagal nerve stimulation can also reduce IS in the absence of heart rate reduction via a mechanism that has been attributed to improvements in mitochondrial function, and attenuation of reactive oxygen species formation, apoptosis and inflammation (reviewed in [52]).

IPC is currently regarded as the gold standard for experimental cardioprotection. Grund et al. demonstrated that increasing the number of cycles of IPC from 1 to 2 progressively decreased regional myocardial oxygen consumption in pigs by 15–25% [44]. Importantly, this reduction in oxygen consumption appears to be due to the reduced energy demand in the myocardium [44]. One of the main limitations of IPC is that it must be applied prior to the injurious ischaemia, so it is limited to predictable ischaemic events. Numerous drugs have been shown to mimic the effects of IPC, but again the question arises of their ability to penetrate and act on the ischaemic region [94, 107]. Remote IPC (RIPC) applied to a limb can be an appealing approach as it can be applied repeatedly and throughout the period of myocardial ischaemia (reviewed in [6]). However, the recent CONDI-2/ERIC-PPCI multicentre trial of RIPC on IS in STEMI patients undergoing primary PCI showed no benefit [46]. Importantly, part of the “remote preconditioning reflex” of RIPC involves activation of the vagal nerve [6, 78]. Consequently, vagal nerve stimulation (VNS) should be able to mimic the effects of RIPC. Indeed, infarct limitation by VNS was demonstrated in an IR model in mice [14]. As the vagal nerve innervates the myocardium, VNS could potentially be used to protect the ischaemic myocardium. It could, therefore, be a potent tool for delaying infarct progression in non-reperfused MI.

In any case, even though techniques to limit oxygen consumption may make it possible to *delay* irreversible myocardial damage within the AAR to a small extent, “death catches up with all of us”, and it is clear that myocardium lacking sufficient oxygen will eventually die. An *increase of oxygen supply* to jeopardised myocardium could potentially preserve its vitality for longer. This could be achieved in two ways: either by an increase of the blood delivery via dilated, pre-existing collaterals, or by an increase of oxygen concentration in blood.

### Strategies to increase oxygen/blood supply

Increasing collateral blood flow could provide significant clinical benefit in patients with coronary artery disease. Collaterals can functionally adapt, increasing blood flow in response to exercise or by pharmacologic stress with adenosine [116]. One the most powerful physiological stimuli for collateral dilation in the setting of acute coronary occlusion is myocardial ischaemia itself [50, 116]. In dog models of MI, acute occlusion of the coronary artery was immediately followed by progressive dilation of collaterals having a diameter less than 100 microns. This response was mediated by ATP-sensitive K^+^ channels [116]. In another study, the vasodilatory effect of acute ischaemia on the microvasculature was mimicked dose-dependently with aprikalim, a selective activator of ATP-sensitive K^+^ channels, and inhibited by glibenclamide [75]. Dilation of collaterals can also be produced by bradykinin or acetylcholine (both partially mediated by NO) [75, 76, 97], or nitroprusside (via direct effects on the smooth muscle) [75]. Regarding the clinical setting, anti-anginal drugs, such as nitroglycerin (glyceryl trinitrate or GTN), clearly provide relief in the setting of myocardial ischaemia. Even the simple application of a GTN patch can reduce IS in mice [126]. Although NO donors, such as GTN, exert their effect primarily by a reduction in cardiac preload, dilation of epicardial coronary arteries also contributes, as well as their selective vasodilator action on well-developed collateral arteries [37, 112]. Unfortunately, a systematic review of three clinical trials showed no consistent evidence of infarct limitation associated with nitric NO treatment as an adjunct to reperfusion [9]. However, the subgroup analysis of a recent multicentre trial showed smaller IS after NO inhalation in GTN-naïve STEMI patients [64]. An interesting way to increase collateral perfusion of the ischaemic myocardium was demonstrated by Jones et al. in a dog model with permanent coronary occlusion [67]. In this study, collateral perfusion in the central and peripheral ischaemic zones of sympathectomised ventricles was 200–400% of those in non-sympathectomised ventricles. As expected, this increase in perfusion was accompanied by reduced IS [67]. However, the IS in this study was evaluated 6 h after coronary ligation, which may indicate a delay in the infarct progression that may not be maintained in the long term [67].

**Fig. 5:**
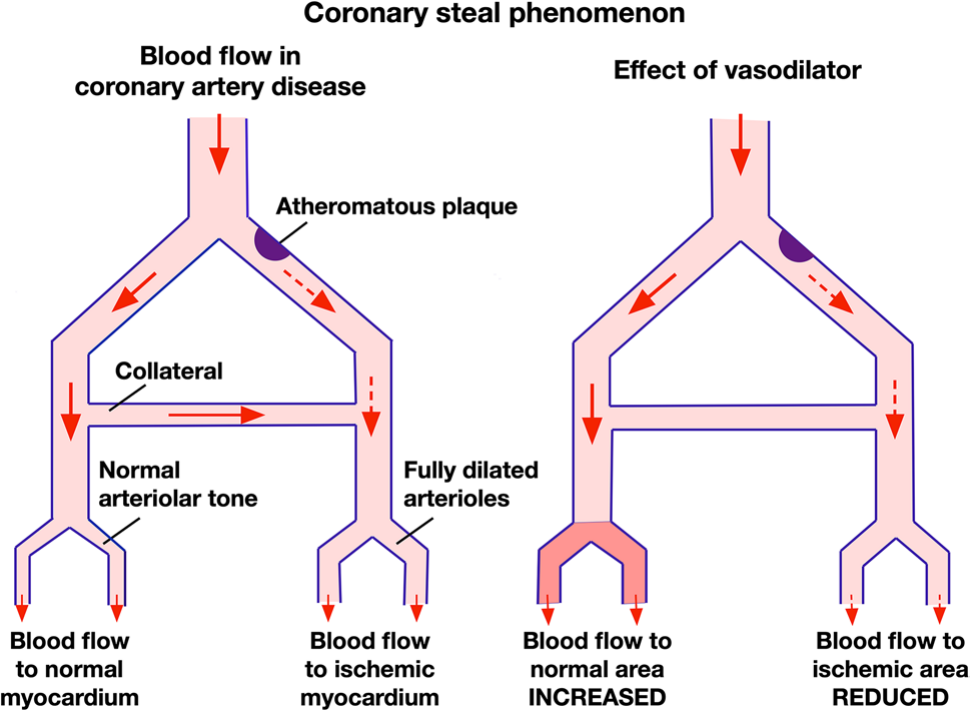
Coronary collateral vessels can allow residual blood flow towards an ischaemic region. In what as known as “coronary steal phenomenon”, an increase in blood flow in a normal region of the myocardium can results in a decrease in blood flow to a partially ischaemic region (here indicated distal to an atheromatous plaque). Redrawn from Ritter et al., Rang & Dale's Pharmacology 8E, 2015:261, with permission from Elsevier

In one experimental study, ventilating anaesthetized mice with pure oxygen instead of room air significantly reduced IS caused by 40-min coronary occlusion followed by 2-h reperfusion [25]. Importantly, ventilation with 100% oxygen in this study was initiated before coronary occlusion. If it is initiated after occlusion, the oxygen would not be able to reach the ischaemic myocardium, as mice lack native coronary collaterals [127]. However, it is known that augmentation of the fraction of inspired oxygen (FiO_2_) does not elevate O_2_ delivery in patients who are not hypoxemic [117]. In addition, increased FiO_2_ has not been found to benefit STEMI patients, possibly due to secondary effects on blood redistribution, as supplemental oxygen causes coronary and systemic vasoconstriction [87, 116]. Vasodilation of coronary vessels could potentially decrease the ischaemic region, either by increasing retrograde blood flow or the diameter of any existing collateral vessels exist. However, in practice, this is complicated by the phenomenon of “coronary steal”, in which dilated coronary vessels paradoxically “steal” the blood away from the ischaemic region (Fig. 5) [7, 116]. One small sign of success was seen in the NACIAM trial combining an antioxidant with nitroglycerin in 112 STEMI patients, although it may have targeted reperfusion rather than ischaemic injury [96].

### Other strategies

One possible therapeutic avenue to targeting ischaemic injury independently of oxygen supply or demand, would be to prevent the calamitous decrease in ATP caused by the FoF1-ATPase. Indeed, FoF1-ATPase inhibitors are extremely effective at slowing ATP depletion and decreasing IS in isolated heats [43, 65]. However, the administration of an FoF1-ATPase inhibitor in vivo is clearly problematic as it will also prevent ATP generation in otherwise healthy cells, and its inhibition must be reversed when oxygen returns to the heart. To overcome these problems, several groups are developing compounds which selectively inhibit the hydrolytic activity of the FoF1-ATPase, but leave its ATP-synthesizing activity intact [43, 62].

Interestingly, the earliest attempts to treat acute MI pharmacologically were performed in the pre-reperfusion era, and were, therefore, intended to target ischaemic injury. The cocktail of glucose–insulin–potassium (GIK) was originally proposed by Sodi-Pallares as a “polarizing” treatment to limit ischaemic injury [115] although we later re-conceptualized it as an activator of the pro-survival PI3-kinase pathway [66]. Unfortunately, clinical trials of GIK provided no conclusive demonstration of its benefit in patients [42].

Other drugs, such as the anti-inflammatory agent, flurbiprofen, were shown to limit IS in dogs subject to CAO, however, we showed that in this case, infarction was only delayed, and not prevented [18]. On the other hand, we did note that this could provide an extended window of opportunity for secondary intervention [18].

## *De novo* coronary collateral formation

Can *de novo* coronary collateral formation prevent ischaemic injury? In clinical practice, a major coronary artery stenosis can sometimes be completely asymptomatic, and is only diagnosed following stress testing and/or angiography. This may be explained by the slow expansion or development over many years of coronary collateral vessels that can supply the under-perfused region, either via remodelling of pre-existing arterial vessels or sprouting of capillaries [50, 63]. Even after STEMI, transmural MI is often avoided in patients with a well-developed network of collaterals. Episodes of ischaemia can also lead to functional adaptation of collaterals [116]. These effects are stimulated via flow-mediated dilatation. Therefore, drugs that cause an increase in endothelial shear stress might increase coronary collateral flow. Indeed, the aforementioned ivabradine appears to increase collateral blood flow [16]. A recent clinical trial of 46 patients with chronic stable coronary artery disease found evidence of improved coronary collateral flow in patients receiving ivabradine for 6 months [39]. Collateral neogenesis can be experimentally induced by gradually increased CAO over a period of many weeks-to-months, and this greatly reduces infarct susceptibility [110, 111]. A study conducted in mice subjected to coronary artery ligation demonstrated relatively rapid *de novo* collateral formation, beginning within 1–2 days and reaching its full extent within 4–7 days [127]. The same time course of collateral growth has been shown for a porcine heart [129]. These studies support data obtained previously by Schaper’s laboratory in different animal species, and can be explained by the experimental observation that the first cellular mitoses occur only 24 h after coronary obstruction [110], which is longer that the survival time of acutely ischaemic muscle. In patients with persistent coronary artery occlusion, as shown by Rentrop et al., the incidence of angiographically demonstrable collaterals increased from 33% at baseline angiography to 90% over 10–14 days [102]. Though new collaterals can be important for cardiac function (reviewed in [63]), as they can counteract further myocardial remodelling, they emerge far too late to be able to salvage cardiomyocytes in the ischaemic area supplied by the occluded coronary artery. Therefore, the only way coronary de novo collateral growth can protect myocardium from ischaemic death caused by chronic CAO, is if their growth is stimulated weeks, months or even years before the ischaemic insult occurs.

## Ventricular remodelling

**Fig. 6:**
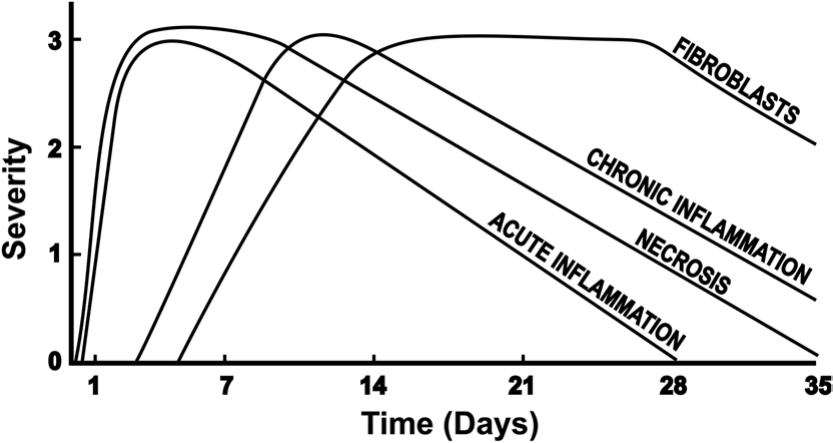
Course of time for histopathologic changes in myocardial infarction in man. Ordinate indicates relative severity of histopathologic changes. Redrawn from Fishbein MC et al. Chest 1978; 73:843–849, with permission from Elsevier

After a prolonged period (weeks) of CAO, the heart undergoes a remodelling process, which can drastically alter its shape and dimensions [57]. As we have seen, ischaemic cardiomyocytes will be long dead by this time, but the remodelling process remains an important therapeutic target [57]. Following CAO, myocardial injury and its hemodynamic consequences initiate systemic neurohormonal activation, which in turn promotes adverse ventricular remodelling. Specifically, this remodelling is manifested as LV dilation and infarct segment lengthening (infarct expansion) [99]. The process of infarct expansion is normally complete within weeks to months, depending on the animal species and medications present. In humans, the first signs of irreversible myocardial injury are already seen within 24 h of symptom onset. However, over time, less necrosis is observed as it is replaced by inflammation, proliferating fibroblasts and deposition of collagen (Fig. 3). The evolution of MI during CAO in rats and mice is more rapid than in humans, possibly due to their smaller body size and higher metabolic rate, associated with higher heart rate [33]. In rats subject to chronic myocardial ischaemia (CAO), a zone of necrosis develops rapidly, becoming transmural (Fig. 2a). Following this, from days 4 to 21, there is gradual shrinkage of the necrotic zone, which coincides with continued prominence of fibroblasts and increased deposition of collagen until the entire necrotic zone is replaced (Fig. 6). The transmural infarction, occupying all the ischaemic area, is replaced by fibrotic tissue, which leads to thinning of the infarcted wall. In most experimental studies, the area of major fibrotic scarring as detected by Masson’s trichrome or Picrosirius Red staining is regarded as being equivalent to the infarcted area [35]. For the evaluation of IS in long-term studies, imaging techniques, such as echocardiography and MRI, have an obvious advantage as they allow dynamic measurements during follow-up. MI can be defined and the size estimated by echocardiography as any segmental wall motion abnormality, such as hypokinesis, akinesis and dyskinesis [95, 108]. In the seminal study by Fishbein et al., 21 days after coronary occlusion, the mean volume of infarcted myocardium in rats was determined by histological staining and was found to decrease from 45.9 to 26.1% of the LV [33]. The multifactorial nature of the remodelling process means that it is complex to quantify “cardioprotection” with a single measure of IS or volume at these later stages.

Prevention of infarct segment lengthening in the setting of non-reperfused MI is essentially equivalent to limiting effective infarct dimensions, and can be achieved by a reduction of wall stress, and/or inhibition of the neurohormonal response. For example, both enalapril and amlodipine administered over 6 weeks limited infarct zone thinning in the non-reperfused groups of dogs [68]. Gourine et al. showed that blockade of beta-adrenoceptors by direct intracerebroventricular administration of metoprolol attenuates the progression of left ventricular remodelling in a rat model of MI-induced heart failure [40]. Thus, alleviating myocardial remodelling in animal models of non-reperfused MI, and specifically, preventing infarct expansion, can mimic the effect of IS reduction to some degree. However, since no cardiomyocytes are really saved from death, this should be seen as a change in infarct morphology rather than a limitation in infarct formation.

One of the major factors contributing to ventricular remodelling is the intense inflammatory response activated by cardiac ischaemia, which can also promote cardiac dysfunction and cardiomyocyte death [57]. As such, inflammation is a potential target for cardioprotection [130]. A key component of this process is NOD-, LRR- and pyrin domain-containing protein 3 (NLRP3)—an intracellular sensor that detects a broad range of danger signals, resulting in the formation and activation of the NLRP3 inflammasome. Assembly of the NLRP3 inflammasome leads to caspase 1-dependent release of the pro-inflammatory cytokines IL-1β and IL-18, as well as to gasdermin D-mediated pyroptotic cell death [119]. In mice and rats with permanent coronary ligation, a marked increase in NLRP3, IL-1β, and IL-18 mRNA expression was found in the LV from 3 to 21 days after CAO, primarily located in myocardial fibroblasts [106]. Caspase-1 activity is increased in the myocardium minutes after the onset of ischaemia and remains elevated for several days, leading to cardiomyocyte death and amplification of inflammation [100]. Mice lacking caspase-1 that were subject to CAO exhibited both improved peri-infarct survival and a decreased rate of ventricular dilatation, presumably due in part to a decrease in MMP-3 activity, IL-18 production, and a reduction in the rate of apoptosis [34]. Likewise, in a non-reperfused MI model, an NLRP3 inhibitor significantly limited LV systolic dysfunction at 7 days [83]. NOD2, an intracellular danger sensor, has also been shown to exacerbate cardiac remodelling in a mouse model, 28 days after permanent coronary occlusion [77]. There was no effect on IS in the above studies. However, in a study by Mezzaroma et al., prevention of inflammasome formation by a P2X7 inhibitor administered immediately after coronary occlusion then daily throughout a 7-day follow-up period, not only limited cardiac enlargement after non-reperfused MI, but also reduced IS [89]. In total, these data show that inflammasome activation following occlusion of the coronary artery causes additional loss of functional myocardium, leading to dilation of the LV and expansion of the infarcted area. Importantly, IS enlargement in this case occurs not only due to myocardium dilation, but possibly also due to the continuing wavefront of myocardial injury although this needs further investigation.

## Myocardial regeneration

Experimentally, many approaches have been investigated to restore functioning cardiomyocytes within the ischaemic zone, although most of these are longer-term prospects and none have yet shown conclusive benefits in patients. These approaches include the myocardial injection of stem or progenitor cells that will differentiate into cardiomyocytes, stimulation of resident stem cells into cardiomyocytes, transdifferentiation of fibroblasts into cardiomyocyte and the application of cardiomyocyte “patches”(reviewed in [80, 81]). As these are still under development, we focus here on myocardial regeneration, which has clearly been demonstrated to occur in neonatal mice.

The adult mammal’s heart is known to be a postmitotic organ. In contrast, cardiomyocytes and non-cardiomyocyte populations in invertebrates regenerate after myocardial damage. The current paradigm is that mammals’ hearts are able to regenerate during the prenatal period, and exposure to atmospheric oxygen in the days after birth causes their heart muscle cells to stop proliferating [17]. However, there is a transition period from a hypoxic intrauterine environment to the postnatal environment with ambient air oxygen. This transition stage has been demonstrated in mice [22], rats [123], and piglets [128]. In these studies, the animals were subjected to permanent coronary artery ligation to create MI within 2 days after birth, followed by complete regeneration of the myocardium within 7–21 days after the ligation. However, the older new-born animals, beginning from the third day after birth, failed to regenerate the infarcted hearts [22, 123, 128]. This ability of a new-born mammal hearts to regenerate has been shown to be attributed to a unique collateral artery development program in neonatal hearts, termed “artery reassembly” [22]. In brief, upon coronary ligation, single arterial endothelial cells migrate as part of the capillary endothelial layer and then coalesce with each other to make collateral arteries [22]. Regarding the mechanism of the mitotic arrest in the postnatal period, it has been suggested that reactive oxygen species-induced oxidative stress is able to arrest the cardiomyocyte cell cycle [92]. Paradoxically, in another study, prolonged hypoxia initiated in two-month-old mice 1 week after the induction of experimental MI, induced reprogramming of adult cardiomyocytes, resulting in re-entry into the cell cycle and heart regeneration [92]. This resulted in decreased myocardial fibrosis following permanent ligation of the left descendant coronary artery. Together, these data suggest that IS resulting from the permanent CAO can be potentially reduced by reprogramming the myocardium adjacent to the infarcted zone into a new-born state.

Interestingly, regeneration of multiple organs in diverse species is known to be guided by nerves, suggesting some evolutionarily conserved nerve functions in regeneration [74]. Specifically, parasympathetic denervation of the neonatal mouse heart either pharmacologically or by mechanical ablation of the vagus nerve, has been demonstrated to reduce cardiomyocyte proliferation and to inhibit heart regeneration following apical resection [82]. Treatment of these denervated mice with nerve-factors rescued the reduced regenerative capacity caused by denervation. Intriguingly, transcriptional profiling of the neonatal mouse heart following vagal denervation revealed disruption of inflammatory gene expression, which was normally activated during heart regeneration. Taken together, these results suggest that cholinergic nerve signalling is required for the proliferation of cardiomyocytes during neonatal mammalian cardiac regeneration [82]. Another study has demonstrated that the profound regenerative capacity of the neonatal mammalian heart requires sympathetic innervation [125]. Here, resection of the left ventricular apex in 2-day-old neonatal mice, was followed by robust re-growth of the sympathetic nerve structures into the regenerating myocardium. Importantly, chemical sympathectomy inhibited sympathetic re-growth and subsequent cardiac regeneration following apical resection [125]. These data indicate the crucial role of nerves in the potential of myocardial regeneration, and suggest that testing for nerve growth markers should not be neglected in the studies witnessing final IS reduction in the models of permanent coronary occlusion.

## Conclusion

In conclusion, in the majority of experimental studies using permanent coronary artery occlusion, the infarct reaches its maximum size within minutes (in animal models without pronounced collateralization), or hours (if extensive collateralization is present). It is, therefore, unrealistic to expect cardioprotective drugs to prevent infarction beyond this time in non-reperfused models of MI. However, it may be possible to *slow* infarct formation by altering oxygen supply and/or utilization. The potential for increasing oxygen supply is extremely limited, especially in animal models lacking coronary collaterals. Importantly, this includes the main experimental models currently used in ischaemia/reperfusion studies: mice, rats and pigs. On the other hand, a reduction in myocardial oxygen demand is achievable, for example by slowing heart rate, mechanical unloading of the LV, endovascular cooling, and potentially IPC or other therapies mimicking IPC. In any case, IS progression can be restrained for only a limited time period [48]. No matter how potent and prolonged the oxygen-sparing therapy, at some point after the coronary occlusion, the reserves of oxygen and metabolic substrates are exhausted, and the cardiomyocytes will inevitably die. Consequently, IS reduction is only possible within hours of permanent occlusion, or when the ischaemic period is followed by reperfusion. Clearly, therefore, any therapy that is directed specifically at delaying the ischaemic damage rather than reperfusion injury should be initiated as soon as possible following occlusion, and before the myocardium is irreversibly damaged. In the ideal scenario, the cardioprotective treatment would be initiated at the very onset of myocardial ischaemia, or even before it. The only situation in which de novo collateral genesis could limit initial infarct formation is if vessel growth is stimulated days or even weeks before the acute coronary occlusion, since the vessels require this time to form.

Can IS be reduced in the days to weeks following permanent coronary occlusion? LV remodelling and fibrotic scar shrinkage may give the impression that the infarct size has reduced, even if there is no increase in cardiomyocyte numbers. One conceivable scenario in which the infarct can be reduced after its formation is with the induction of myocardial regeneration. Since this would also require the proliferation of new coronary vessels and nerves in addition to cardiomyocytes, these all require careful evaluation in experimental studies of chronic coronary occlusion when IS reduction via regeneration is being proposed. Currently, awakening the endogenous mechanisms of heart regeneration is viewed as an achievable therapeutic target, but many challenges remain (reviewed in [13]).

Finally, in experimental studies relying on histochemical and immunohistochemical methods to detect and measure the infarcted area, the IS can only be evaluated once, at the end of the experiment. Conversely, MRI, which is currently widely used even in small animal species, can be used repeatedly for infarct measurement, and has clear benefits in allowing true IS reduction to be distinguished from regeneration and remodelling. In addition, it can allow evaluation of an approximate area at risk (although this remains challenging). The use of modern imaging techniques to re-investigate some of the therapeutic agents that have previously demonstrated IS reduction in non-reperfused animal models may shed some light on their mechanism of infarct limitation.
